# Effects of The Legend of Zelda: Breath of the Wild and Studio Ghibli Films on Young People’s Sense of Exploration, Calm, Mastery and Skill, Purpose and Meaning, and Overall Happiness in Life: Exploratory Randomized Controlled Study

**DOI:** 10.2196/76522

**Published:** 2025-08-01

**Authors:** Annisa Arigayota, Barbara Duffek, Congcong Hou, Andreas Benedikt Eisingerich

**Affiliations:** 1 Imperial College Business School Imperial College London London United Kingdom; 2 Robinson College of Business Georgia State University Atlanta, GA United States; 3 Faculty of Commerce Kyushu Sangyo University Fukuoka Japan

**Keywords:** open-world games, nostalgia, lab experimental study, postgraduate students, My Neighbor Totoro, Kiki’s Delivery Service, Nintendo

## Abstract

**Background:**

Young people feel increasingly anxious and sad nowadays. Engaging with works of art and entertainment, such as playing open-world games or watching Studio Ghibli films, can be more than just a pastime. However, the extent to which, if at all, open-world games and feelings of nostalgia affect overall happiness in life remains unclear.

**Objective:**

This study aimed to examine the extent to which open-world games, such as *The Legend of Zelda: Breath of the Wild*, and nostalgia evoked by Studio Ghibli films, such as Hayao Miyazaki’s *My Neighbor Totoro* or *Kiki’s Delivery Service*, affect postgraduate students’ sense of exploration, calm, mastery and skill, purpose and meaning, and, ultimately, happiness in life.

**Methods:**

A controlled laboratory experiment was conducted using a 2 (playing an open-world game vs no open-world game) × 2 (nostalgia vs no nostalgia) between-subject design. Study participants (N=518) were randomly assigned to the study’s 4 conditions and answered a brief questionnaire, examining their sense of exploration, calm, mastery and skill, purpose and meaning, and, ultimately, happiness in life. As part of the study, we conducted univariate analysis and bootstrapping-based moderated mediation analysis with 5000 resamples.

**Results:**

The results showed a significant and positive impact of playing an open-world game on overall life happiness (mean [M]_playedgame_ 4.563, SD 0.072, vs M_notplayedgame_ 3.170, SD 0.072; F_(1, 517)_=117.246, *P*<.001). Furthermore, the positive impact of open-world games on overall life happiness was significantly enhanced by nostalgia evoked by watching Studio Ghibli films (M_nostalgia_ 5.45, SD 0.102, vs M_nonostalgia_ 3.58, SD 0.102; SE 0.144, 95% CI 1.332-1.900; *P*<.001). Moreover, exploratory moderated mediation with bootstrapped-based analyses and 5000 resamples demonstrated that the effect of playing open-world games on happiness is mediated by a sense of exploration (effect=0.11; SE 0.05, 95% CI 0.04-0.21), sense of calm (effect=0.32; SE 0.09, 95% CI 0.15-0.51), sense of skill and mastery (effect=0.08; SE 0.05, 95% CI 0.01-0.18), and sense of purpose and meaning (effect=0.32; SE 0.14, 95% CI 0.06-0.60).

**Conclusions:**

This study shows that playing an open-world game, such as The *The Legend of Zelda: Breath of the Wild*, and nostalgia evoked by Studio Ghibli films significantly foster a sense of exploration and calm in life, as well as a feeling of mastery and skill, and purpose and meaning, hence ultimately contributing positively to one’s overall happiness in life.

**Trial Registration:**

ISRCTN ISRCTN14757739; https://www.isrctn.com/ISRCTN14757739

## Introduction

### Overview of Young People’s Anxiety and Reduced Happiness

Prior work indicates that young people feel increasingly stressed, anxious, and sad [1-5]. Many educational systems have moved toward heightened testing, earlier introduction of complex curriculum, and an emphasis on standardized, measurable achievements [6-8]. Young people can feel pressured to perform well on standardized exams from a young age, which can foster a fear of failure and chronic stress [6,9]. Even outside of formal testing, parents and others often place a strong emphasis on high achievements, such as getting top grades or excelling in extracurricular activities [6,10]. Although setting goals can be positive, an overemphasis on competition rather than joy in the activity per se can lead to children developing anxiety and burnout. In addition, a growing number of young people have daily schedules packed with lessons, sports, and various extracurricular activities [2]. Although these can be rewarding, they often leave little room for imaginative free play or downtime, which are crucial elements of healthy emotional development and stress relief [2].

Being constantly guided or supervised by adults can limit a young person’s ability to develop decision-making skills and self-confidence [2]. As a result, they may feel anxious when they do not have control or when they encounter new situations without adult direction [2,11]. Furthermore, young people nowadays have unprecedented access to social media, where they often may face cyberbullying, peer pressure, information overload, or unrealistic portrayals of others’ lives [2,12]. Constant comparison to curated online images can breed insecurity and anxiety [2,12,13]. Further, from a young age, many children also hear about worldwide crises, such as pandemics, natural disasters, extreme violence, and climate change, through online 24/7 news cycles, television, or social media [14-17]. This constant influx of alarming information can create a sense of fear, disorientation, hopelessness, and uncertainty about the world [17].

Moreover, young people often mirror the emotional climate of their household [18,19]. If parents are under significant financial or job-related stress, children may internalize those anxieties [20-23]. Moreover, shifts in the family structure, such as divorce, separation, or frequent relocations, can lead to insecurity and uncertainty, which contributes to elevated stress levels in young people [24-28]. Further, parents’ best intentions, such as trying to protect children from hardship, can sometimes lead to “helicopter” or “tiger” parenting [10,29-33]. Often, children are expected to not only do well in school but also develop artistic talents, athletic achievements, and social popularity. The perceived need to “excel at everything” can be a significant source of anxiety [30,33] and existential worries in young people who, in essence, are still learning how to process big-picture problems [34].

### Overview of Open-World Games, Nostalgia, and Happiness in Life

Playing open-world video games, such as *The Legend of Zelda: Breath of the Wild*, can have several positive effects on an individual’s happiness and overall well-being. For instance, open-world games give players a high degree of control over their actions and how they experience the game world [35,36]. Moreover, the vast, interactive landscapes in *The Legend of Zelda: Breath of the Wild* encourage exploration, which can be deeply satisfying [35,37]. This mirrors the human desire for discovery and adventure, providing a sense of wonder and joy that can be rare in daily life.

Further, open-world video games offer an escape from everyday stressors [38-42]. The calming visuals of Hyrule, combined with the game’s music and the freedom to roam, can be soothing, acting as a form of stress relief. In addition, the act of playing can require full attention, akin to flow, where players are fully engaged in the present moment, which can reduce anxiety and increase happiness. Moreover, playing open-world games, such as *The Legend of Zelda: Breath of the Wild* provides clear goals (eg, freeing the Divine Beasts or completing shrines, collecting Korok Seeds), which can give players a sense of purpose and progress, elements known to enhance life satisfaction. Furthermore, players can approach problems in *The Legend of Zelda: Breath of the Wild* in multiple ways. Players often create their own stories or ways to interact with the game world, giving them a platform for personal narrative and expression.

Nostalgia is often characterized as a bittersweet emotional experience that arises when reflecting on memories from the past—typically those that carry personal significance or recall “the good old days” [43-45]. Nostalgia can affect people’s happiness and overall well-being in several ways [43,44,46]. For instance, nostalgia can help people remember meaningful and treasured episodes in their lives, reinforcing values, personal milestones, and who they are at their core. This may offer a sense of continuity and grounding in a world that is often seen as increasingly complex and rapidly changing [44,47]. In times of stress, reflecting on cherished memories can foster optimism and hope [48,49]. Nostalgia may, thus, also increase a sense of belonging and connection, as reminiscing about events that a group or community experienced can strengthen group identity and foster unity. When people use nostalgic memories to celebrate who they have become and to highlight their strengths and resources, it may help boost self-esteem and gratitude in times of personal challenges and difficulty [48]. Reflection can become a catalyst for personal growth and contentment if channelled in a way that supports forward momentum. That is, nostalgia can be a comforting, identity-affirming experience that deepens social ties and personal meaning. Therefore, nostalgia can be a valuable tool for reaffirming one’s sense of meaning and purpose [49].

Studio Ghibli films, such as works by Hayao Miyazaki (*My Neighbor Totoro* and *Kiki’s Delivery Service*), may have a unique capacity to evoke nostalgia and foster a sense of happiness in viewers of all ages. The reasons behind this phenomenon range from the richly detailed animation and gentle storytelling to the deeper themes of community, family, and profound connection to nature. Films such as *My Neighbor Totoro* and *Kiki’s Delivery Service* often focus on the small, everyday wonders of life, such as playing in the countryside or sharing mealtime with family. By shining a spotlight on ordinary moments and turning them into something magical, Miyazaki’s works tap into a universal longing for the innocence and wonder of childhood. This kind of warmth can evoke nostalgia for times when people felt safe and cared for.

Although prior work shows heightened levels of anxiety, sadness, and depression among young people, questions surrounding how open-world games, such as *The Legend of Zelda: Breath of the Wild*, and nostalgia, evoked by Studio Ghibli films, such as Miyazaki’s *My Neighbor Totoro* and *Kiki’s Delivery Service*, may impact young people’s happiness in life remain largely unanswered. Thus, in this study, we aimed to answer the following research question: How does playing an open-world game and nostalgia evoked by watching a Studio Ghibli film impact young people’s sense of exploration, calm, mastery and skill, purpose and meaning in life, and, ultimately, overall life happiness?

### Background on Open-World Games and Nostalgia Evoked by Studio Ghibli Films

#### Effects on Happiness in Life

**Figure 1:**
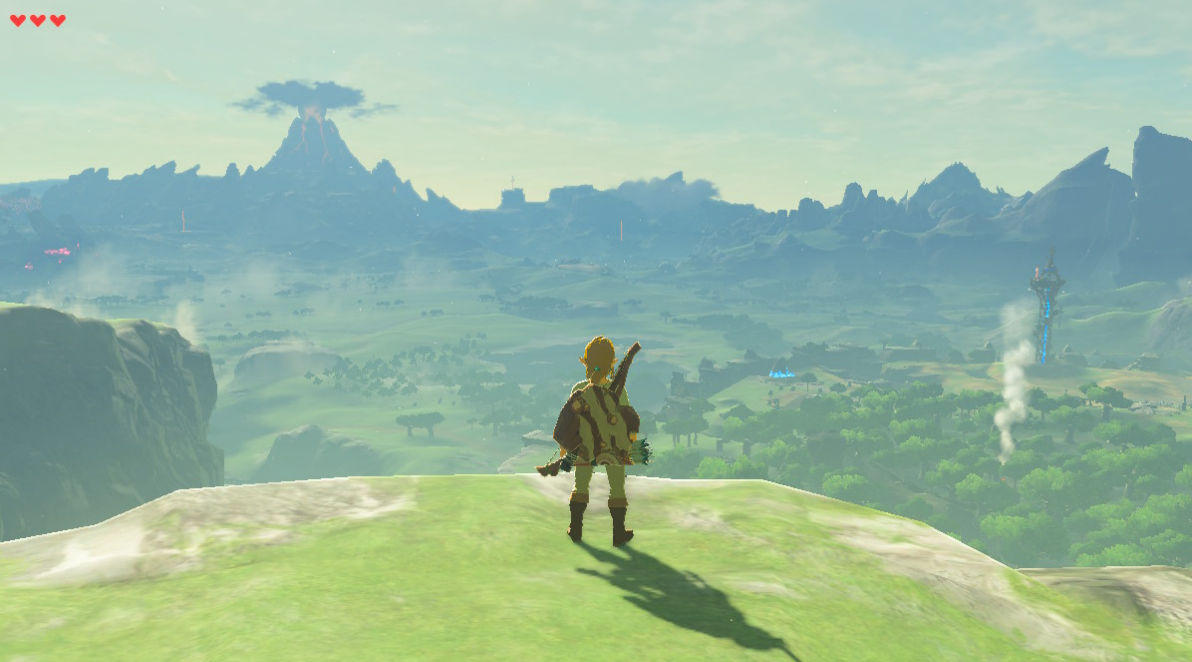
Open-world landscape in *The Legend of Zelda: Breath of the Wild* (from Nintendo).

One of the most praised elements of open-world games, such as *The Legend of Zelda: Breath of the Wild*, is their vast, open-world landscape. Being able to freely roam lush forests, climb mountains, or glide across canyons provides a relaxing form of escapism (Figure 1). The freedom to chart one’s own path fosters a sense of agency and control, which can be especially soothing in times of real-life stress or uncertainty [35,50]. Furthermore, the environments of *The Legend of Zelda: Breath of the Wild* are filled with tranquil music, detailed natural landscapes, and a subdued color palette. Stopping by a quiet pond or cooking a meal under the stars in-game may momentarily transport the player away from daily stresses, letting them recharge mentally. In addition, although there is a main storyline, the game places few constraints on when or how one progresses. This encourages players to set their own pace. The act of wandering without immediate pressure to win can be liberating, as it allows for relaxation and personal goal setting rather than constant performance demands.

**Figure 2:**
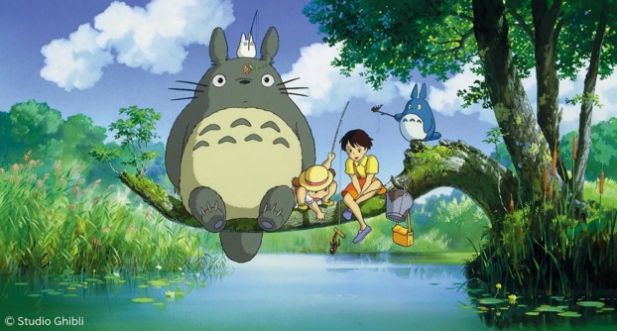
A scene of nature in *My Neighbor Totoro* (with permission from Studio Ghibli).

At the core of many Studio Ghibli films is a message of uplifting harmony, whether it is living peacefully alongside nature (as in *My Neighbor Totoro*; Figure 2) or finding your place in a new community (as in *Kiki’s Delivery Service*). These uplifting themes can be deeply comforting and can remind viewers of simple joys and quiet wonders in life. Studio Ghibli stories often highlight the extraordinary within the ordinary and, hence, put emphasis on everyday life’s magic. This perspective can encourage viewers to find small delights and moments of beauty in their own routines.

Both open-world games, such as *The Legend of Zelda: Breath of the Wild*, and nostalgia evoked by Studio Ghibli films may inspire childlike wonder and a sense of discovery. For instance, *The Legend of Zelda: Breath of the Wild* frequently rewards curiosity. Stumbling upon hidden shrines, secrets, or quirky nonplayer characters creates a constant cycle of discovery. This taps into a childlike sense of wonder, where every cliff might hide a new vista and every cave might hold a surprise. Further, activities such as collecting ingredients, cooking new recipes, or just standing at a high vantage point to appreciate varied weather patterns and admire a scenic view encourage a slow and mindful approach to gameplay.

**Figure 3:**
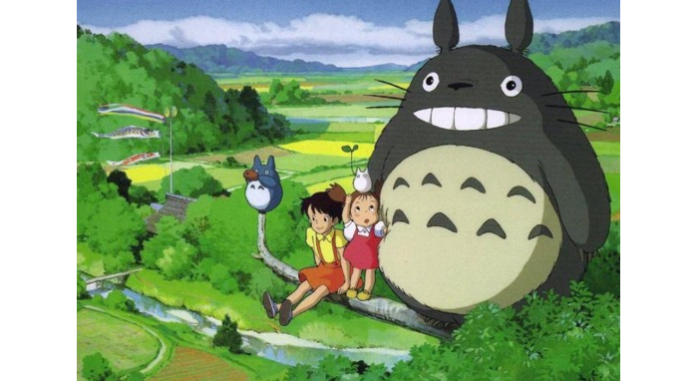
Depiction of fields and forests in *My Neighbor Totoro* (with permission from Studio Ghibli).

A hallmark of Studio Ghibli films is the breathtaking portrayal of nature—the rolling hills, forests, and gardens that appear onscreen (Figure 3). Nature in these films is not just a backdrop; it is a character in its own right. Seeing characters treat the environment with reverence and kindness fosters a sense of harmony. This connection can remind viewers of the comfort and grounding effect that comes from being in tune with the natural world—a feeling many associate with childhood adventures and freedom.

#### Effects on a Sense of Exploration

Hyrule in *The Legend of Zelda: Breath of the Wild* includes deserts, snow-capped mountains, tropical beaches, forests, and more. Each region has unique landscapes, weather patterns, and resources. The breathtaking vistas and day-night/weather cycles make exploration intrinsically rewarding. Standing on a mountaintop at sunrise to overlook Hyrule fosters a feeling of awe and accomplishment. Furthermore, the presence of 900 Korok Seeds rewards the smallest inklings of curiosity, whether it is lifting a seemingly random rock, diving off a peak into a hidden ring, or examining a peculiar formation of objects. These frequent, bite-size surprises reinforce an exploratory mindset, teaching players that small detours often lead to pleasant discoveries.

Studio Ghibli films open windows to worlds where magic coexists with ordinary life, inviting audiences to explore both physical and emotional landscapes with fresh eyes. By grounding fantastical elements in relatable, slice-of-life experiences, viewers are encouraged to look for wonder in the ordinary. Studio Ghibli films often center on young protagonists, such as Mei and Satsuki in *My Neighbor Totoro* and Kiki in *Kiki’s Delivery Service*. Their openness to discovery and excitement about new surroundings convey a sense of unfiltered curiosity. This perspective invites audiences, adults included, to reconnect with childlike wonder and remember what it felt like to explore the unknown with fresh enthusiasm. Rather than focusing on large-scale spectacles, these films linger on small, intimate moments. By spotlighting the beauty in small details, Studio Ghibli’s storytelling fosters an appreciation for the little wonders in day-to-day life, reminding viewers that exploration is not always about epic quests; indeed, it can be about noticing the world right in front of us.

Moreover, a hallmark of Miyazaki’s work is the deep reverence for nature. Trees, fields, rivers, clouds, and skies are lovingly portrayed as living, breathing elements. Studio Ghibli films subtly remind us that adventure can be found anywhere if we approach life with curiosity. This focus on venturing into the unknown and embracing challenges conveys the idea that exploration is both external (navigating a new environment) and internal (discovering who you are). Critically, these gentle, wonder-filled narratives do not emphasize grandiose heroics. Instead, they celebrate quieter forms of exploration, including making friends or learning how to navigate a new space. It is a reminder that exploration is often less about the destination and more about an attitude of open-minded curiosity. In doing so, they can inspire viewers to become explorers in their own lives, seeking out the hidden pockets of wonder that exist all around us.

#### Effects on a Sense of Calm

Open-world games provide a structured yet boundless space to take a break from real-life stressors. When individuals are immersed in Link’s journey and the vastness of Hyrule, daily frustrations may feel more distant. This timeout can give people a chance to process emotions and reset. Watching a sunrise in the game, sheltering from a rainstorm, or stargazing at night can feel almost meditative. These small, ambient details anchor players to calm moments and encourage them to pause and appreciate the virtual world. Moreover, *The Legend of Zelda: Breath of the Wild* allows individuals to tackle challenges in multiple ways, often rewarding creative problem solving more than raw skill. People can bypass certain obstacles or find clever solutions using runes. This flexibility reduces the frustration that can come from being stuck in a linear progression. In addition, the soft, often minimalistic soundtrack uses gentle piano and nature sounds, fostering a peaceful ambiance that can help soothe an angry or anxious mind.

**Figure 4:**
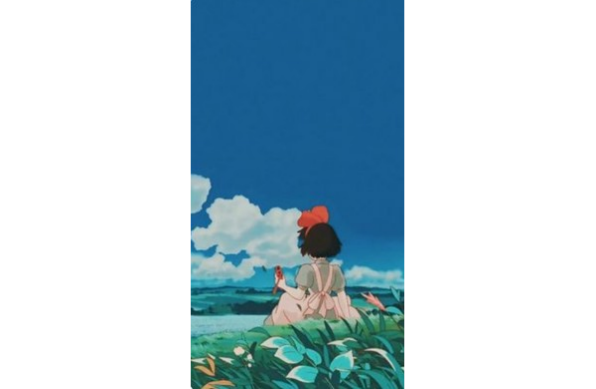
Depiction of Kiki looking at clouds in *Kiki’s Delivery Service* (with permission from Studio Ghibli).

Even when confronted with difficulties—a lost broom, a sick parent, or initial loneliness in a new town, Studio Ghibli’s young heroes maintain hope and kindness. Seeing them overcome these challenges encourages a positive mind set in viewers, reminding them of their own capacity for resilience. Studio Ghibli narratives are often propelled by acts of empathy, such as neighbors helping neighbors, spirits offering aid, and strangers becoming friends. In *My Neighbor Totoro*, for instance, the forest spirits show concern for the sisters, and the father’s understanding approach to parenting enriches the film’s sense of warmth. This kindhearted environment fosters a safe, comforting world that puts emphasis not on overdramatization of emotions but on kindness as a driving force (Figure 4).

#### Effects on a Sense of Mastery and Skill

Earning better weapons, upgrading one’s armor, and increasing one’s “hearts” or stamina wheel visibly reflect the effort put in by a player. This tangible growth fosters a feeling of skill advancement, as players see Link literally become stronger. Finding a Korok Seed, cooking a successful new recipe, or completing a shrine are all microrewards. Collectively, these frequent small wins build a consistent loop of effort→reward, reinforcing a sense of competence. In *The Legend of Zelda: Breath of the Wild*, players face numerous challenges that require problem solving, perseverance, and creativity. The game normalizes “failing forward,” such as dying to a Lynel, and then refining strategies. This mirrors Kiki’s journey of overcoming self-doubt and the sisters’ adventures in *My Neighbor Totoro* to find their mother. These narratives collectively teach resilience, adaptability, and the value of personal growth, which are integral to finding purpose and sustaining happiness. Both gaming and the films celebrate learning from one’s mistakes. This can encourage individuals to view their life’s challenges as opportunities for development rather than setbacks.

#### Effects on a Sense of Purpose and Meaning

Open-world games, such as *The Legend of Zelda: Breath of the Wild*, and Studio Ghibli films may do more than entertain; they can offer players a profound sense of purpose and meaning.

*The Legend of Zelda: Breath of the Wild*’s classic “hero’s journey” storyline resonates with deeply rooted myths about overcoming adversity. Engaging with these archetypal narratives can prompt a sense of mission: players feel they are contributing to a cause bigger than themselves, even if it is within a fictional world. This “bigger than self” feeling can be deeply meaningful. Furthermore, many players find calm and meditative moments in the game by simply riding through open fields, quietly gathering resources, or watching sunrises atop tall peaks. These peaceful interludes can trigger introspection and help players engage with inner thoughts. The sense of wonder generated can remind people that there is more to life than daily stressors, instilling renewed purpose.

**Figure 5:**
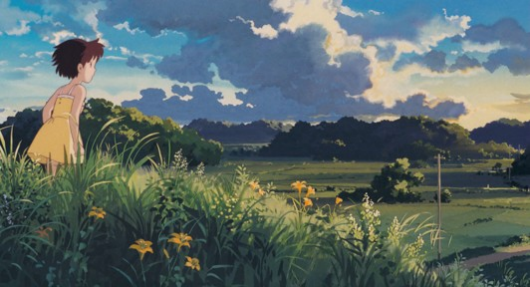
Depiction of Satsuki looking for her young sister in *My Neighbor Totoro* (with permission from Studio Ghibli).

**Figure 6:**
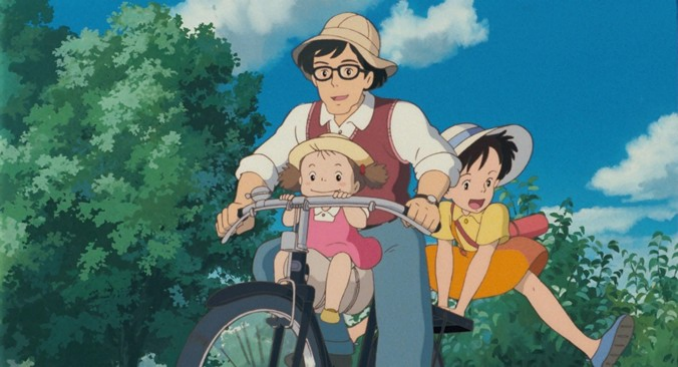
Depiction of ricing a bicycle together in *My Neighbor Totoro* (with permission from Studio Ghibli).

Instead of intense battles, Studio Ghibli films often focus on internal struggles (eg, Kiki’s loss of confidence, Satsuki’s and Mei’s anxiety about their mother’s health; Figure 5). In so doing, the stories highlight how purpose emerges from the personal growth that comes from confronting self-doubt, fear, or sorrow. Studio Ghibli protagonists find value in simple daily tasks (Figure 6), delivering goods, tidying up a house, or taking care of siblings. These ordinary moments become opportunities to strengthen relationships, contribute to the community, and experience quiet joy. This gentle reminder that purpose can be found in common routines, by embracing curiosity, wonder, and a respect for the unseen or unexplored aspects of life, may encourage viewers to reassess the significance of their own everyday actions.

Hence, through intimate storytelling, focus on personal growth, and a compassionate depiction of community and nature, Studio Ghibli films gently guide viewers toward a greater awareness of what truly matters in life. By showcasing characters who discover meaning in acts of kindness, self-discovery, and a loving relationship with the world around them, these films inspire audiences to seek out or rekindle their own sense of purpose, whether through connection with others, appreciation of the every day, or a renewed embrace of their own unique gifts.

## Methods

### Study Design

The completed CONSORT-SPI (Consolidated Standards of Reporting Trials for Social and Psychological Interventions) 2018 checklist for reporting randomized trials of social and psychological interventions is shared in Multimedia Appendix 1. We registered our randomized control study with the International Standard Randomised Controlled Trial Number (ISRCTN) registry (ISRCTN 14757739). More specifically, as part of this study, we used a 2 (playing an open-world game vs no open-world game) × 2 (nostalgia vs no nostalgia) between-subject experimental design. In spring 2025, posters and flyers were used on a university campus to recruit postgraduate university students to take part in a study on daily activities and well-being over the course of 3 weeks. All study participants were randomly allocated to 1 of the 4 study conditions using a random number generator:

- Condition 1: playing an open-world game + nostalgia
- Condition 2: playing an open-world game + no nostalgia
- Condition 3: no open-world game + nostalgia
- Condition 4 (control group): no open-world game + no nostalgia

We conducted the study in a lab experimental setting on a university campus. In condition 1, participants were invited to spend 30 minutes to play *The Legend of Zelda: Breath of the Wild* on the Nintendo Switch in handheld mode. After 30 minutes of video gameplay, the participants were invited to watch a brief 7-minute clip from a Studio Ghibli film (randomly assigned so that some participants watched *My Neighbor Totoro* and others watched *Kiki’s Delivery Service*). Finally, the participants completed a brief questionnaire.

In condition 2, participants were invited to spend 30 minutes to play the open-world game as in Condition 1. After having played the game for 30 minutes, participants in condition 2 completed the brief questionnaire.

In condition 3, participants were randomly allocated to watch a brief 7-minute clip from either Studio Ghibli’s *My Neighbor Totoro* or *Kiki’s Delivery service* and subsequently complete a brief questionnaire.

Finally, in condition 4 (control group), participants simply completed the brief questionnaire.

### Ethical Considerations

Ethical approval was received from Kyushu Sangyo University’s Ethics Committee (approval number 2024-0017). All study participants were informed that the data collected will only be used to inform academic research. Further, they were reassured that data will be anonymized and treated with strict confidentiality. In addition, participants were told that they are free to stop and withdraw from the study at any time and without giving a reason. They were also informed that the data from participants who stop early will not be used as part of the study. Written consent forms were obtained from all study participants after they were offered an information leaflet, and time (minimum of 24 hours) for consideration of whether to take part in the study was allowed. Each study participant was thanked and received US $5 as a token of gratitude.

### Participants and Procedure

A total of 530 university postgraduate students took part in this study. Twelve participants decided to stop and withdraw from the study during the data collection process. The data from the participants who decided to withdraw were not used as part of the study analyses. Hence, we obtained an effective total number of 518 (97.7%) participants for the study (condition 1: n=129, 24.9%; condition 2: n=130, 25.1%; condition 3: n=130, 25.1%; condition 4: n=129, 24.9%). Across all 4 conditions, participants answered a brief questionnaire, with responses scored on a 9-item Likert scale (from 1=strongly disagree to 9=strongly agree) capturing participants’ overall life happiness; sense of exploration, calm, mastery and skill, and purpose and meaning in life; and the manipulation check questions for nostalgia.

As controls, we captured participants’ enjoyment of the video game in conditions 1 and 2 (“I very much enjoyed playing the video game,” “I found playing the video game to be interesting”; r=0.76), enjoyment of the Studio Ghibli film clip in conditions 1 and 3 (“I enjoyed watching the film clip,” “I did not like the film clip very much” [reverse coding]; r=0.71), familiarity with the video game in conditions 1 and 2 (“I am very familiar with the video game,” “I know the story of this video game very well”; r=0.81), and familiarity with the film in conditions 1 and 3 (“I am very familiar with the characters in the film,” “I know the story of the characters in the film very well”; r=0.77), with anchors 1=strongly disagree to 9=strongly agree. The controls did not affect the results and thus are not discussed further.

### Statistical Analysis

We checked measurement items and support for convergent validity and specifically examined whether the estimates for the average variance extracted (AVE) were indeed higher than the recommended threshold of 0.50 [51,52]. In addition, we examined the possibility that measurement errors may vary across items and calculated and compared the AVE for all pairs of constructs under investigation to the squared correlation between the 2 constructs of interest [53]. Moreover, we examined Cronbach α and composite reliability (CR) values. We conducted ANOVA as part of our manipulation checks for nostalgia and univariate analyses using IBM SPSS Statistics version 29.0. To examine the potential synergistic effect of nostalgia on the effects of open-world games on a sense of exploration, calm, skill and purpose, and overall happiness in life, we conducted an exploratory bootstrapping-based moderated mediation analysis (model 8) with 5000 resamples [54].

## Results

### Data Analysis

**Figure 7:**
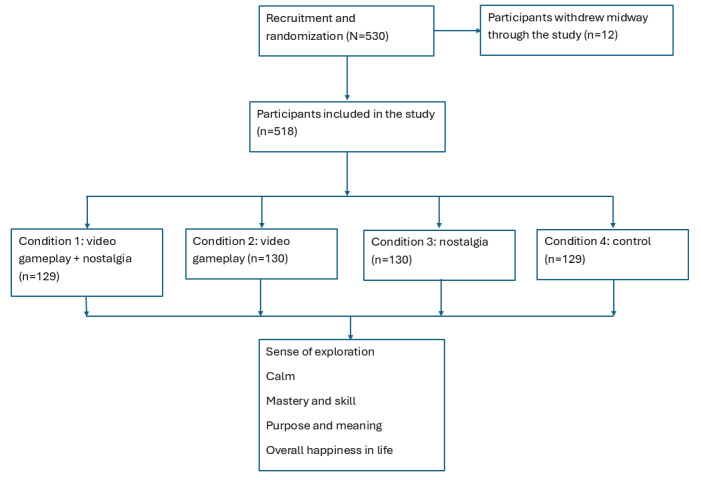
Study flowchart.

Figure 7 shows the participant selection flowchart.

Analysis results showed that the AVE exceeded the recommended threshold of 0.50 [51,52]. Moreover, the squared correlation between any pair of constructs was not higher than the respective AVE for each of the constructs in the pair, in support of discriminant validity [53]. In addition, Cronbach α and CR values were high. Detailed measurement items are listed in Table 1.

**Table 1:** Study measurement items and reliabilities.

Variable	Measure	Cronbach α	AVE^a^	CR^b^
Happiness in life	“I feel grateful for the good things in my life.” “I am satisfied with the overall direction of my life and look forward to what lies ahead.” “Overall, I would describe myself as a happy person.” “I generally feel a sense of peace and contentment when I think about my life as a whole.”	.90	0.59	0.85
Sense of exploration	“I appreciate seeking out new experiences, even if they feel unfamiliar or slightly uncomfortable at first.” “I view my life as an adventure that lies ahead, filled with possibilities and opportunities.” “I view each day as a chance to discover something new.”	.92	0.78	0.91
Sense of calm	“I feel calm and collected in my daily life.” “I am feeling angry about a lot of things in my life.” (Reverse-coded) “I generally feel upset.” (Reverse-coded)	.93	0.75	0.90
Sense of skill and mastery	“I can find solutions when I face difficulties in my life.” “I believe I am well equipped to navigate life’s day-to-day challenges.” “I have the necessary skills to master life.”	.94	0.85	0.95
Sense of purpose and meaning	“I feel my life contributes to something larger than myself.” “I experience a deep sense of fulfilment when I think about the path I am on.” “Overall, I view my life as meaningful and purposeful.”	.95	0.86	0.95
Manipulation check for nostalgia	“I feel a longing for how things used to be.” “Memories from my past bring me comfort and warmth.” “I get sentimental thinking about the ‘good old days’.”	.93	0.84	0.94

^a^AVE: average variance extracted.

^b^CR: composite reliability.

### Manipulation Checks

A 2 × 2 ANOVA on the manipulation check confirmed that the manipulation of nostalgia was indeed successful. As expected, participants in the nostalgia-induced conditions indicated higher feelings of nostalgia (mean [M]_nostalgia_ 6.61, SD 1.87) compared to those in conditions where nostalgia was not induced (M_nonostalgia_ 3.77, SD 2.15; *F*_(1, 517)_=257.38, *P*<.001). No significant effects of playing the video game (*F*_(1, 517)_=0.124, *P*=.73) or game × nostalgia interaction (*F*_(1, 517)_=0.582, *P*=.45) were observed.

### Cell Means and Univariate Analyses

This study explored the positive effect of playing open-world games on overall happiness (M_playedgame_ 4.563, SD 0.072, vs M_notplayedgame_ 3.170, SD 0.072; *F*_(1, 517)_=117.246, *P*<.001). The study results also showed a significant and positive impact of nostalgia evoked by watching Studio Ghibli films, such as Miyazaki’s *My Neighbor Totoro* and *Kiki’s Delivery Service* on happiness. Playing video games had a positive impact on happiness, and the effect was enhanced when nostalgia was induced (M_nostalgia_ 5.45, SD 0.102, vs M_nonostalgia_ 3.58, SD 0.102; SE=0.144, 95% CI 1.332-1.900, *P*<.001). Tables 2-5 present univariate analysis results and cell means comparing the effects.

**Table 2:** Results on happiness (dependent variable): study cell means estimates.

Video game played	Nostalgia induced	Mean (SD)	SE (95% CI)
No	No	2.409 (0.98)	0.102 (2.208-2.610)
No	Yes	3.931 (1.08)	0.102 (3.731-4.131)
Yes	No	3.579 (1.18)	0.102 (3.379-3.779)
Yes	Yes	5.547 (1.37)	0.102 (5.345-5.748)

**Table 3:** Effect of video gameplay on happiness (dependent variable): cell means comparisons (based on estimated marginal means).

Video game played	Nostalgia (I)	Nostalgia (J)	Mean difference (I – J)	SE (95% CI)	*P* value
No	No	Yes	–1.522^a^	0.144 (–1.806 to –1.238)	<.001
Yes	No	Yes	–1.968^a^	0.144 (–2.251 to –1.684)	<.001

^a^The mean difference was significant at the .05 level.

**Table 4:** Effect of nostalgia on happiness (dependent variable): cell means comparisons (based on estimated marginal means).

Nostalgia	Video game played (I)	Video game played (J)	Mean difference (I – J)	SE (95% CI)	*P* value
No	No	Yes	–1.170^a^	0.144 (–1.454 to –0.886)	<.001
Yes	No	Yes	–1.616^a^	0.144 (–1.900 to –1.332)	<.001

^a^The mean difference was significant at the .05 level.

**Table 5:** Results on happiness (dependent variable): univariate test results.

Condition			Sum of squares		Mean square		*F*_(1, 514)_		*P* value
**Video game not played**									
	Contrast	149.951		149.961		111.011		<.001	
	Error	694.344		1.351		—^a^		—	
**Video game played**									
	Contrast	250.689		250.689		185.577		<.001	
	Error	694.344		1.351		—		—	
**Nostalgia not induced**									
	Contrast	88.625		88.625		65.606		<.001	
	Error	694.344		1.351		—		—	
**Nostalgia induced**									
	Contrast	169.035		169.035		125.131		<.001	
	Error	694.344		1.351		—		—	

^a^Not applicable.

### Exploratory Moderated Mediation

To examine the effect of nostalgia on playing video games and happiness in life, we conducted an exploratory bootstrapping-based moderated mediation analysis with 5000 resamples (model 8) [54]. The analysis was conducted with happiness as the dependent variable; whether the participant played or did not play a video game as the independent variable; a sense of exploration, calm, skill and mastery, and purpose and meaning as mediating variables; and whether nostalgia was or was not induced as the moderator variable. The sample size was 518. Detailed results are presented in Tables 6-18. The detailed results showed that the effect of playing video games on happiness is mediated by a sense of exploration (effect=0.11, SE=0.05, 95% CI 0.04-0.21; Tables 6-8), a sense of calm (effect=0.32, SE=0.09, 95% CI 0.15-0.51; Tables 9-11), a sense of skill and mastery (effect=0.08, SE=0.05, 95% CI 0.01-0.18; Tables 12-14), and a sense of purpose and meaning (effect=0.32, SE=0.14, 95% CI 0.06-0.60; Tables 15-17). Table 18 shows the means for key constructs across the 4 study conditions. Together, these results highlight the importance of playing video games and nostalgia on happiness, through a sense of exploration, calm, skill and mastery, and purpose and meaning.

**Table 6:** Regression results with a sense of exploration as a mediator.

Dependent variable and predictors		B	SE (95% CI)	*t* Test (*df*)	*P* value
**Sense of exploration (R=0.64, R²=0.40, mean standard error=2.47, *F*_(3, 514)_=116.28, *P*<.001)**					
	Constant	1.24	0.68 (–0.09 to 2.57)	1.83 (514)	.07
	Video game played	0.39	0.43 (–0.44 to 1.24)	0.93 (514)	.36
	Nostalgia	–0.12	0.43 (–0.97 to 0.72)	–0.29 (514)	.77
	Video game played × nostalgia	1.06	0.27 (0.53 to 1.59)	3.93 (514)	<.001
**Happiness (R=0.70, R²=0.49, mean standard error=1.33, *F*_(4, 513)_=124.87, *P*<.001)**					
	Constant	0.03	0.51 (–0.97 to 1.03)	0.06 (513)	.95
	Video game played	0.68	0.32 (0.05 to 1.31)	2.13 (513)	.03
	Sense of exploration	0.11	0.03 (0.04 to 0.17)	3.19 (513)	.002
	Nostalgia	1.09	0.32 (0.46 1.72)	3.40 (513)	.001
	Video game played × nostalgia	0.33	0.21 (–0.07 to 0.74)	1.63 (513)	.11

**Table 7:** Conditional effects of playing a video game on happiness at levels of nostalgia.

Nostalgia induced	Effect	SE (95% CI)	*t* Test (*df*)	*P* value
No	1.02	0.15 (0.72-1.31)	6.73 (513)	<.001
Yes	1.35	0.17 (1.03-1.68)	8.15 (513)	<.001

**Table 8:** Conditional indirect effects of playing a video game on happiness via a sense of exploration.^a^

Nostalgia induced	Effect	BootSE (95% CI)
No	0.15	0.05 (0.06-0.27)
Yes	0.27	0.08 (0.11-0.44)

^a^Index of moderated mediation: index=0.11, BootSE=0.0450 (95% CI 0.04-0.21).

**Table 9:** Regression results with a sense of calm as a mediator.

Dependent variable and predictors		B	SE (95% CI)	*t* Test (*df*)	*P* value
**Sense of calm (R=0.71, R²=0.51, mean standard error=2.62,*F*_(3, 514)_=175.46, *P*<.001)**					
	Constant	0.09	0.71 (–1.31 to 1.48)	0.13 (514)	.90
	Video game played	0.22	0.45 (–0.66 to 1.11)	0.49 (514)	.62
	Nostalgia	1.03	0.45 (0.14 to 1.91)	2.78 (514)	.02
	Video game played × nostalgia	1.08	0.28 (0.52 to 1.64)	3.79 (514)	<.001
**Happiness (R=0.76, R²=0.57, mean standard error=1.12, *F*_(4, 513)_=172.01, *P*<.001)**					
	Constant	0.14	0.47 (–0.78 to 1.05)	0.29 (513)	.77
	Video game played	0.66	0.29 (0.08 to 1.24)	2.24 (513)	.03
	Sense of calm	0.30	0.03 (0.24 to 0.36)	10.37 (513)	<.001
	Nostalgia	0.77	0.30 (0.19 to 1.35)	2.50 (513)	.01
	Video game played × nostalgia	0.13	0.19 (–0.25, to 0.49)	0.65 (513)	.51

**Table 10:** Conditional effects of playing a video game on happiness at levels of nostalgia (mediator: sense of calm).

Nostalgia induced	Effect	SE (95% CI)	*t* Test (*df*)	*P* value
No	0.78	0.14 (0.51-1.05)	5.71 (513)	<.001
Yes	0.90	0.15 (0.61-1.19)	6.09 (513)	<.001

**Table 11:** Conditional indirect effects of playing a video game on happiness via a sense of calm.^a^

Nostalgia induced	Effect	BootSE (95% CI)
No	0.49	0.07 (0.26-0.54)
Yes	0.71	0.10 (0.52-0.93)

^a^Index of moderated mediation: index=0.32, BootSE=0. 0903 (95% CI 0.15-0.51).

**Table 12:** Regression results with a sense of skill and mastery as a mediator.

Dependent variable and predictors		B	SE (95% CI)	*t* Test (*df*)	*P* value
**Sense of skill and mastery (R=0.50, R²=0.25, mean standard error=3.28, *F*_(3, 514)_=58.23,*P*<.001)**					
	Constant	2.73	0.80 (1.16 to 4.29)	3.42 (514)	<.001
	Video game played	–0.54	0.50 (–1.53 to 0.45)	–1.07 (514)	.28
	Nostalgia	–1.13	0.50 (–2.12 to –0.14)	–2.25 (514)	.03
	Video game played × nostalgia	1.47	0.32 (0.84 to 2.09)	4.61 (514)	<.001
**Happiness (R=0.70, R²=0.49, mean standard error=1.34, *F*_(4, 513)_=121.94, *P*<.001)**					
	Constant	0.01	0.51 (–1.01 to 1.02)	0.01 (513)	.99
	Video game played	0.76	0.32 (0.12 to 1.39)	2.34 (513)	.02
	Sense of calm	0.06	0.03 (0.00 to 0.11)	2.02 (513)	.04
	Nostalgia	1.14	0.33 (0.50 to 1.78)	3.42 (513)	<.001
	Video game played × nostalgia	0.36	0.21 (–0.04 to 0.77)	1.74 (513)	.08

**Table 13:** Conditional effects of playing a video game on happiness at levels of nostalgia (mediator: sense of skill and mastery).

Nostalgia induced	Effect	SE (95% CI)	*t* Test (*df*)	*P* value
No	1.12	0.15 (0.83-1.40)	7.63 (513)	<.001
Yes	1.48	0.16 (1.17-1.79)	9.30 (513)	<.001

**Table 14:** Conditional indirect effects of playing a video game on happiness via a sense of skill and mastery.^a^

Nostalgia induced	Effect	BootSE (95% CI)
No	0.05	0.03 (0.00-0.11)
Yes	0.14	0.07 (0.01-0.27)

^a^Index of moderated mediation: index=0.08, BootSE=0.0448 (95% CI 0.01-0.18).

**Table 15:** Regression results with a sense of purpose and meaning as a mediator.

Dependent variable and predictors		B	SE (95% CI)	*t* Test (*df*)	*P* value
**Sense of purpose and meaning (R=0.39, R²=0.16, mean standard error=4.02, *F*_(3, 514)_=31.89, *P*<.001)**					
	Constant	1.64	0.88 (–0.09 to 3.73)	1.86 (514)	.06
	Video game played	–0.21	0.56 (–1.31 to 0.88)	–0.38 (514)	.71
	Nostalgia	0.18	0.56 (–0.92 to 1.28)	0.32 (514)	.75
	Video game played × nostalgia	0.79	0.35 (0.10 to 1.49)	2.25 (514)	.02
**Happiness (R=0.86, R²=0.74, mean standard error=0.69, *F*_(4, 513)_=358.40, *P*<.001)**					
	Constant	–0.50	0.37 (–1.01 to 1.02)	–1.37 (513)	.17
	Video game played	0.81	0.23 (0.12 to 1.39)	3.51 (513)	<.001
	Sense of calm	0.41	0.02 (0.00 to 0.11)	22.19 (513)	<.001
	Nostalgia	1.00	0.23 (0.50 to 1.78)	4.35 (513)	<.001
	Video game played × nostalgia	0.12	0.15 (–0.04 to 0.77)	0.84 (513)	.40

**Table 16:** Conditional effects of playing a video game on happiness at levels of nostalgia (mediator: sense of purpose and meaning).

Nostalgia induced	Effect	SE (95% CI)	*t* Test (*df*)	*P* value
No	0.94	0.10 (0.73-1.14)	9.01 (513)	<.001
Yes	1.06	0.11 (0.85-1.27)	9.97 (513)	<.001

**Table 17:** Conditional indirect effects of playing a video game on happiness via a sense of purpose and meaning.^a^

Nostalgia induced	Effect	BootSE (95% CI)
No	0.23	0.09 (0.06-0.42)
Yes	0.57	0.11 (0.34-0.78)

^a^Index of moderated mediation: index=0.32, BootSE=0.14 (95% CI 0.06-0.60).

**Table 18:** Means across study conditions (N=518).

Variable	Conditions, mean (SD)			
	Open-world game + Studio Ghibli film (nostalgia)	Open-world game	Studio Ghibli film (nostalgia)	Control group
Happiness in life	5.55^a^ (1.37)	3.58^b^ (1.18)	3.93^c^ (1.08)	2.41^d^ (.98)
Sense of exploration in life	6.04^a^ (1.73)	4.03^c^ (1.41)	3.51^b^ (1.47)	2.57^d^ (1.53)
Sense of calm in life	6.90^a^ (1.85)	3.72^b^ (1.45)	4.52^c^ (1.73)	2.42^d^ (1.39)
Sense of skill and mastery in life	5.26^a^ (2.06)	3.45^c^ (1.54)	2.86^b^ (1.83)	2.53^b^ (1.79)
Sense of purpose and meaning in life	4.74^a^ (2.52)	2.98^c^ (1.92)	3.37^c^ (1.69)	2.40^b^ (1.79)
Manipulation check for nostalgia	6.71^a^ (1.73)	3.73^c^ (2.22)	6.51^a^ (2.01)	3.80^c^ (2.08)

^a-d^Means with different superscripts are significantly different at *P*<.05.

## Discussion

### Principal Findings

Studio Ghibli films, such as *My Neighbor Totoro* and *Kiki’s Delivery Service*, alongside open-world games, such as *The Legend of Zelda: Breath of the Wild*, offer immersive, interactive experiences that cultivate happiness by fostering wonder and exploration, calm, a sense of mastery and skill, and a sense of purpose and meaning in life. These works blend storytelling, exploration, and emotional engagement to nurture a sense of purpose and joy. These works are not mere distractions; our findings demonstrate that they may be viewed as “active escapism” that restores energy. This study shows how they can synergistically enhance young people’s sense of exploration, as well as feelings of calm, mastery and skill, and purpose and meaning.

### Comparison With Prior Work

Previous research has offered important insights into the role of video games in people’s lives [55,56] and that multiplayer games can mitigate loneliness during social isolation [57,58], while excessive play may reduce mental health [59,60]; however, critical questions remain about how open-world games may affect people’s overall happiness in life. Although prior studies have linked open-world games to cognitive escapism and relaxation benefits [35] and video games to well-being [61] and improved mood [62,63] and have noted nostalgia as a coping mechanism during stressful times [64-66] that enhances meaning and continuity [45,67-70], this study examined the synergistic effects of open-world games and nostalgia evoked by Studio Ghibli films on happiness in life. In addition, this study examined the process mechanism that helps explain the influence of open-world games, such as *The Legend of Zelda: Breath of the Wild*, and nostalgia evoked by Studio Ghibli films on life happiness. More specifically, this study makes a novel contribution by showing how open-world games and Studio Ghibli films can synergistically enhance people’s overall happiness in life through a sense of exploration; feelings of calm, mastery, and skill; and a sense of purpose and meaning.

Specifically, this study shows that Studio Ghibli, such as *My Neighbor Totoro* and *Kiki’s Delivery Service*, and open-world games, such as *The Legend of Zelda: Breath of the Wild*, do not just entertain, they offer frameworks for living well. They encourage us to seek wonder in the ordinary (eg, Totoro’s acorns, Korok puzzles), embrace challenges as growth (eg, Kiki’s flight, Link’s battles), and find strength in community and solitude alike (eg, Satsuki’s bond with Mei, Link’s lone journey). By blending imaginative storytelling with interactive agency, Studio Ghibli films and open-world games can provide a holistic recipe for happiness: one part mindfulness and calm, one part mastery of skill and purpose, and endless curiosity and exploration. As Kiki learns to fly again and Link gazes at Hyrule’s horizon, these stories whisper, “Happiness is not a destination, it is how we travel.”

### Limitations

This study has a number of limitations, which offer promising avenues for future research. First, we examined an expressed sense of exploration, calm, mastery and skill, purpose and meaning, and life happiness in a cross-sectional manner. Additional research that investigates the long-term effects of open-world games and nostalgia on actual life happiness, actual exploration, calm, skill, and purpose in life is richly deserving. Second, we accounted for participants’ familiarity with and enjoyment of the open-world game in conditions 1 and 3 in our study, but we did not capture their immersion in the game and feelings of flow across our study conditions. We invite future research to account for immersion, flow, cognitive escapism, and relaxation in the study of open-world games and nostalgia. Third, we studied postgraduate students in one cultural setting. We encourage further study of playing open-world games and Studio Ghibli films across different countries and study participants to account for other potential confounding effects, including participants’ gender, age, cultural background, and lifestyles. Fourth, we studied the effects of 2 Studio Ghibli films. To enhance confidence in the generalizability of the results, we encourage future research to examine the effects of other Studio Ghibli films, such as *Castle in the Sky* and *Howl’s Moving Castle*, on people’s sense of nostalgia and happiness in life.

### Future Research

Prior research indicates that in addition to the mediators investigated in this study, there may be other avenues through which open-world gameplay and nostalgia can affect people’s happiness in life [71-75]. For instance, people may get attached to the games they play and the stories they engage with [76-81], which may offer them comfort and enhance happiness in a world of turmoil. Moreover, feelings of mastery and skill, as well as elevation and inspiration, have been noted to positively affect people’s lives [82-85]. We encourage future research to further unpack the roles of open-world gameplay and nostalgia in affecting people’s satisfaction and long-term happiness. For instance, additional research that examines how open-world games and interaction with stories, such as Studio Ghibli films, may offer experiences seen as authentic, enrich people’s lives, reduce feelings of loneliness and psychological pain, and help them walk through life with purpose, energy, joy, and fulfilment is richly deserving. By showing how open-world games, such as *The Legend of Zelda: Breath of the Wild*, and Studio Ghibli films enhance people’s overall happiness in life in a synergistically manner, this study bridges gaming and film culture with mental health science. In so doing, the study challenges outdated stereotypes about gaming and engaging with storytelling and opens doors to innovative, accessible tools for well-being. We invite additional research to examine the potential of video gameplay and storytelling in affecting people’s lives. This study indicates that what is so beautiful about Studio Ghibli films and *The Legend of Zelda: Breath of the Wild* is that they are not just tales or games but are also invitations—invitations to see the world with wider eyes, to stumble into hidden meadows of joy, and to remember that even when life feels like a tangled *Lost Woods*, there is always a path (or a paraglider) waiting to be discovered. They whisper, never forget, “You are the hero of your own Hyrule, the witch flying her own broom, and the kid who still knows how to wave at the Catbus.” Future research exploring the potential influence of video gameplay and digital solutions in enhancing childlike wonder and the overall happiness in life and in reducing the risk of burnout is richly deserving.

### Conclusion

This controlled laboratory experiment (N=518) shows the positive effects open-world gameplay and nostalgia can have by reducing postgraduate students’ stress, anxiety, and burnout. The findings of this study show how participants playing open-world games, such as *The Legend of Zelda: Breath of the Wild*, and watching Studio Ghibli films, such as Miyazaki’s *My Neighbor Totoro* and *Kiki’s Delivery Service*, may report increased happiness, as well as increased feelings of exploration, calm, mastery and skill, and purpose and meaning in life.

## Appendix

Multimedia Appendix 1 CONSORT-SPI (Consolidated Standards of Reporting Trials for Social and Psychological Interventions) 2018 checklist.

## Abbreviations

AVE: average variance extracted
CR: composite reliability

## Data Availability

Data are available from the authors upon reasonable request.
